# Bioinformatics analysis reveals the potential target of rosiglitazone as an antiangiogenic agent for breast cancer therapy

**DOI:** 10.1186/s12863-022-01086-2

**Published:** 2022-09-16

**Authors:** Adam Hermawan, Herwandhani Putri

**Affiliations:** 1 Laboratory of Macromolecular Engineering, Department of Pharmaceutical Chemistry, Faculty of Pharmacy, Universitas Gadjah Mada Sekip Utara II, Yogyakarta, 55281 Indonesia; 2 Cancer Chemoprevention Research Center, Faculty of Pharmacy, Universitas Gadjah Mada Sekip Utara II, Yogyakarta, 55281 Indonesia

**Keywords:** Rosiglitazone, Breast cancer, Angiogenesis, Bioinformatics, Targeted therapy

## Abstract

**Background:**

Several studies have demonstrated the antitumor activity of rosiglitazone (RGZ) in cancer cells, including breast cancer cells. However, the molecular targets of RGZ in the inhibition of angiogenesis in breast cancer cells remain unclear. This study aimed to explore the potential targets of RGZ in inhibiting breast cancer angiogenesis using bioinformatics-based analysis.

**Results:**

Venn diagram analysis revealed 29 TR proteins. KEGG pathway enrichment analysis demonstrated that TR regulated the adipocytokine, AMPK, and PPAR signaling pathways. Oncoprint analysis showed genetic alterations in *FABP4* (14%), *ADIPOQ* (2.9%), *PPARG* (2.8%), *PPARGC1A* (1.5%), *CD36* (1.7%), and *CREBBP* (11%) in patients with breast cancer in a TCGA study. The mRNA levels of *FABP4*, *ADIPOQ*, *PPARG*, *CD36*, and *PPARGC1A* were significantly lower in patients with breast cancer than in those without breast cancer. Analysis of gene expression using bc-GenExMiner showed that the mRNA levels of *FABP*, *ADIPOQ*, *PPARG*, *CD36, PPARGC1A*, and *CREBBP* were significantly lower in basal-like and triple-negative breast cancer (TNBC) cells than in non-basal-like and non-TNBC cells. In general, the protein levels of these genes were low, except for that of CREBBP. Patients with breast cancer who had low mRNA levels of *FABP4*, *ADIPOQ*, *PPARG*, and *PPARGC1A* had lower overall survival rates than those with high mRNA levels, which was supported by the overall survival related to DNA methylation. Correlation analysis of immune cell infiltration with TR showed a correlation between TR and immune cell infiltration, highlighting the potential of RGZ for immunotherapy.

**Conclusion:**

This study explored the potential targets of RGZ as antiangiogenic agents in breast cancer therapy and highlighted FABP4, ADIPOQ, PPARG, PPARGC1A, CD36, and CREBBP as potential targets of RGZ. These findings require further validation to explore the potential of RGZ as an antiangiogenic agent.

**Supplementary Information:**

The online version contains supplementary material available at 10.1186/s12863-022-01086-2.

## Highlights

- Recent studies have focused on the development of indirect angiogenesis inhibitors.
- Bioinformatics-based identification of potential rosiglitazone target genes to inhibit breast cancer angiogenesis.
- FABP4, ADIPOQ, PPARG, PPARGC1A, CD36, and CREBBP are potential targets of rosiglitazone.

**Supplementary Information:**

The online version contains supplementary material available at 10.1186/s12863-022-01086-2.

## Background

Angiogenesis or neovascularization is the growth of new blood vessels in body tissues that are required by cancer cells to meet their nutrient intake, oxygen, and waste disposal needs for the tumor mass to continue growing and spreading [1]. Angiogenesis allows cells to receive nutrients and oxygen for survival [2]. Cancer initiation, invasion, and metastasis are angiogenesis-dependent events [3]. Most angiogenic also act as anti-metastatic [4].

Angiogenesis inhibitors are divided into two classes: direct and indirect inhibitors [5]. Direct angiogenesis inhibitors, such as canstatin, angiostatin, and tumstatin, directly target endothelial cells and prevent microvascular endothelial cells from responding to various angiogenic proteins, thus inhibiting proliferation, migration of endothelial cell and avoiding cell death [6]. Indirect angiogenesis inhibitors, including tyrosine kinase inhibitors typically block the expression of tumor proteins that trigger angiogenesis or stop their activity, as well as suppress the expression of their receptors in endothelial cells [7].

A peroxisome proliferator-activated receptor-gamma (PPAR) agonist called rosiglitazone (RGZ) is clinically used to treat type 2 diabetes mellitus (T2DM) [8]. Several previous studies have demonstrated the antitumor activity of RGZ in cancer cells, including breast cancer cells [8]. RGZ also increased the sensitivity of MDA-MB 231 cells to tumor necrosis factor-alpha, CH11, and CYC202 [8]. Clinical trials of RGZ early stage breast cancer patients have shown that PPARγ signaling is activated in breast cancer cells [9].

Previous studies have demonstrated that RGZ prevents the growth and angiogenesis of endothelial cells; therefore, it has the potential to be employed as an atherosclerosis treatment [10]. Other studies have shown that the antiangiogenic activity of RGZ in human umbilical vein endothelial cells is mediated by the opening of maxi-K channels due to the activation of PPARγ by RGZ [11]. Another study showed that RGZ inhibits angiogenesis in chick chorioallantoic membranes and endothelial cell migration [12]. A randomized controlled trial of RGZ in humans showed that RGZ reduced adipocyte size and increased capillary density and serum adiponectin levels [13]. RGZ inhibits angiogenesis in myeloma cells by regulating PI3K/Akt and ERK signaling pathways [14]. However, the molecular targets of RGZ in the inhibition of angiogenesis in breast cancer (BC) cells remain unclear.

**Fig. 1:**
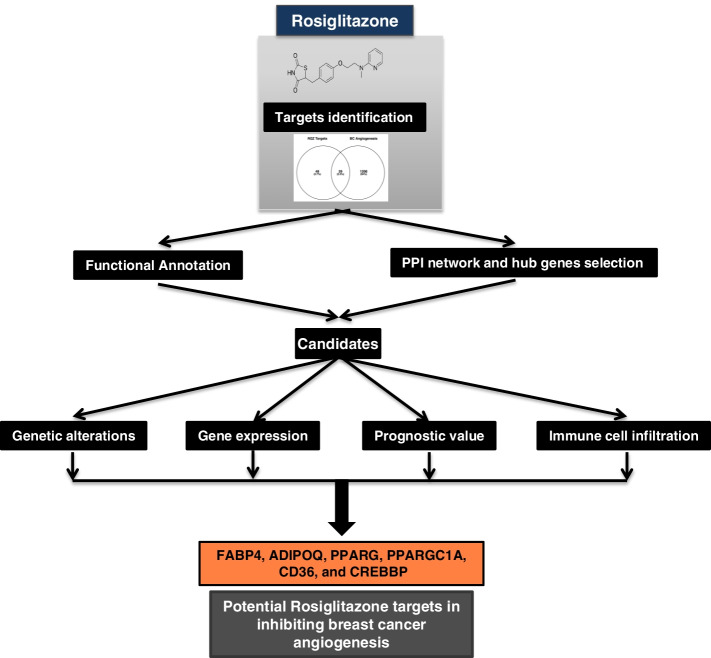
Flowchart of the study

This study aimed to investigate the potential RGZ target genes in inhibiting breast cancer angiogenesis using bioinformatics-based analysis (Fig. 1). RGZ protein targets were retrieved from the STITCH and STRING publicly available databases, and RGZ potential target genes in angiogenesis inhibition (TR) were identified by analyzing Venn diagrams with breast cancer angiogenesis regulatory genes. Functional annotation of TR, protein–protein interaction (PPI) network, hub gene selection, genetic alteration, and DNA methylation analyses, and KM plots were performed to uncover the potential targets of RGZ in inhibiting angiogenesis. The results of this study could serve as a basis for the development of targeted breast cancer therapy using RGZ to inhibit angiogenesis.

## Methods

### Data preparation

Direct target proteins (DTPs) from RGZ were obtained from STITCH (http://stitch.embl.de/) [15] based on the default settings from the website. Indirect target proteins (ITPs) from each DTP were retrieved from STRING (https://string-db.org/) version 11.0 [16], with a confidence score setting of 0.4, and the maximum amount of interactions to show was no more than 10. Breast cancer angiogenesis regulatory genes were obtained from OMIM (https://www.omim.org/) [17] with the keywords “breast cancer angiogenesis” and “homo sapiens,” and gene symbols were selected.

### Analysis of PPI network and selection of hub genes

PPI network visualization was performed using GENEMANIA (https://genemania.org/) [18] under default settings from the database. Hub genes were selected using Cytoscape version 3.7.1 and CytoHubba plugin [19] based on degree methods in accordance with the default settings from the database.

### Functional annotation of the TR

Functional annotation of the TR was performed using ShinyGO v0. 75 (http://bioinformatics.sdstate.edu/go/) using default database settings [20]. Gene ontology assesments of including biological processes, cellular components, and molecular functions, and pathway enrichment network analysis were performed with Fisher’s exact test, using a *p* value < 0.05, as a threshold for significance.

### Analysis of genetic alterations in selected TR

Genetic alterations analysis in selected TR were conducted using cBioportal (https://www.cbioportal.org/) [21, 22]. In brief, the selected TR (as a gene symbol) was submitted as a query to the database and genetic alterations were searched for among breast cancer studies. The breast cancer study with the highest amount of genetic alterations was selected for Oncoprint analysis to determine the type of alterations among breast cancer samples. A one-way ANOVA with Tukey’s multiple comparison test was used to statistically examine the number of genetic changes in each gene. Mutual exclusivity analysis was performed to explore the mutual alterations among TR gene pairs of TR by Fisher’s exact test. Statistical significance was set at *p*-value < 0.05.

### DNA methylation analysis of selected TR

To ascertain the expression and prognostic patterns of single CpG methylation of *FABP4, ADIPOQ, PPARG, PPARGC1A, CD36*, and *CREBBP* in breast cancer, we used MethSurv (https://biit.cs.ut.ee/methsurv/ [23]. DNA methylation values were depicted in this analysis using beta values (beta values ranging from 0 to 1). The M/(M + U + 100) equation was used to calculate each CpG methylation. The intensity values M and U were methylated and unmethylated, respectively, as previously described [24].

### Analysis of gene expression in selected TR

Gene expression was analyzed using GEPIA to determine the expression of selected TR in breast cancer cells and adjacent tissues (http://gepia.cancer-pku.cn/) [25] under default settings from the database. The method for differential analysis was one-way ANOVA. Statistical significance was set at *p*-value < 0.01. Targeted expression analysis of selected TR was performed using Breast Cancer Gene Expression Miner v4.5 (bc-GenExMiner v4.5) (http://bcgenex.centregauducheau.fr). In brief, the selected TR was submitted as a gene symbol and searched in the RNA-seq data of TCGA samples from a population of basal-like and triple-negative breast cancer (TNBC) [26]. The differences in gene expression among the different population groups were analyzed using Welch’s test. Statistical significance was set at *p*-value < 0.01.

### Protein expression in selected TR

The Human Protein Atlas (HPA, https://www.proteinatlas.org/) was used to determine the protein levels of FABP4, ADIPOQ, PPARG, PPARGC1A, CD36, and CREBBP in healthy and malignant breast tissues [27, 28].

### Kaplan–Meier survival analysis

The prognostic value of TR expression in breast cancer was analyzed using the Kaplan–Meier survival curve from KMPlotter (https://kmplot.com/) based on overall survival (OS) [29]. Statistical significance was set at *p*-value < 0.05. The prognostic value of a single CpG of TR in patients with breast cancer was analyzed using the MethSurv database, and the threshold of significance was a likelihood ratio (LR) test, with *p*-value < 0.05 [23, 24].

### Correlation analysis of immune cell infiltration with TR

The correlation of TR with immune cell infiltration was calculated using the TIMER 2.0 database (http://timer.comp-genomics.org/) [30]. Spearman’s correlation coefficient was used to perform the correlation analysis. An inverse correlation is shown by a negative score, whereas a positive value shows a direct association. A value< 0.05 was considered significant.

## Results

### Data preparation

**Fig. 2:**
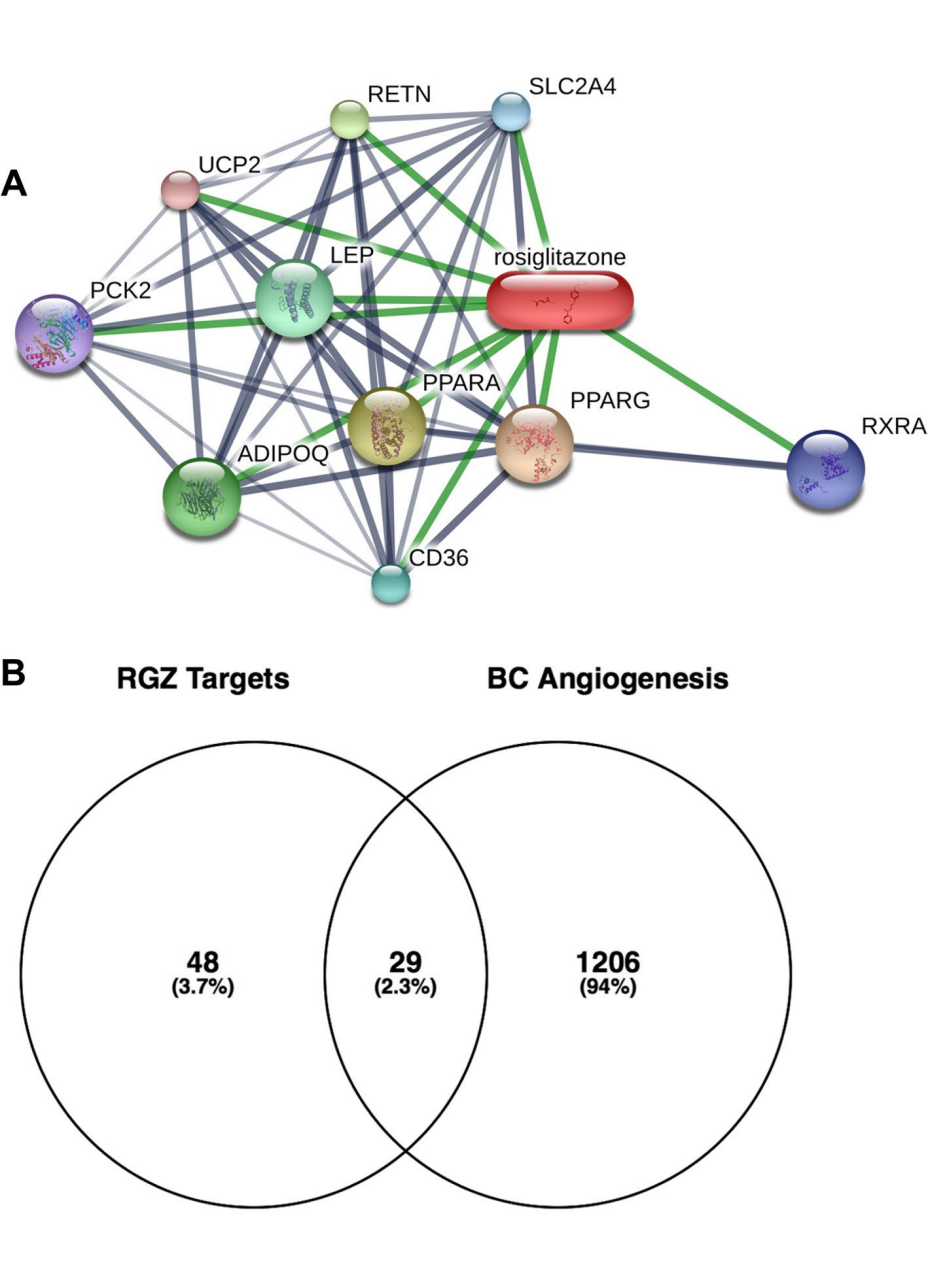
**A** Interaction between RGZ and its direct target proteins (DTPs), as analyzed using STITCH. **B** Venn Diagram analysis between RGZ targets and breast cancer (BC) angiogenesis regulatory genes, resulting in potential target of RGZ against angiogenesis (TR)

DTPs of RGZ were retrieved from STITCH, yielding 10 proteins: PPARG, PPARA, CD36, RXRA, ADIPOQ, PCK2, UCP2, RETN, SLC2A4, and LEP (Fig. 2A). From each DTP, ITPs were searched for using STRING and 67 ITPs were identified (Supplementary Table 1). All proteins targeted by RGZ, consisting of 10 DTPs and 67 ITPs, were considered RGZ targets. The angiogenesis regulatory gene was obtained from OMIM and produces 1235 regulators, which is referred to as BC angiogenesis (Supplementary Table 2). Analysis of the Venn diagram yielded 29 protein targets that could be potential RGZ targets in inhibiting breast cancer angiogenesis (TR) (Fig. 2B, Supplementary Table 3).

### Analysis of PPI network and selection of hub proteins

**Fig. 3:**
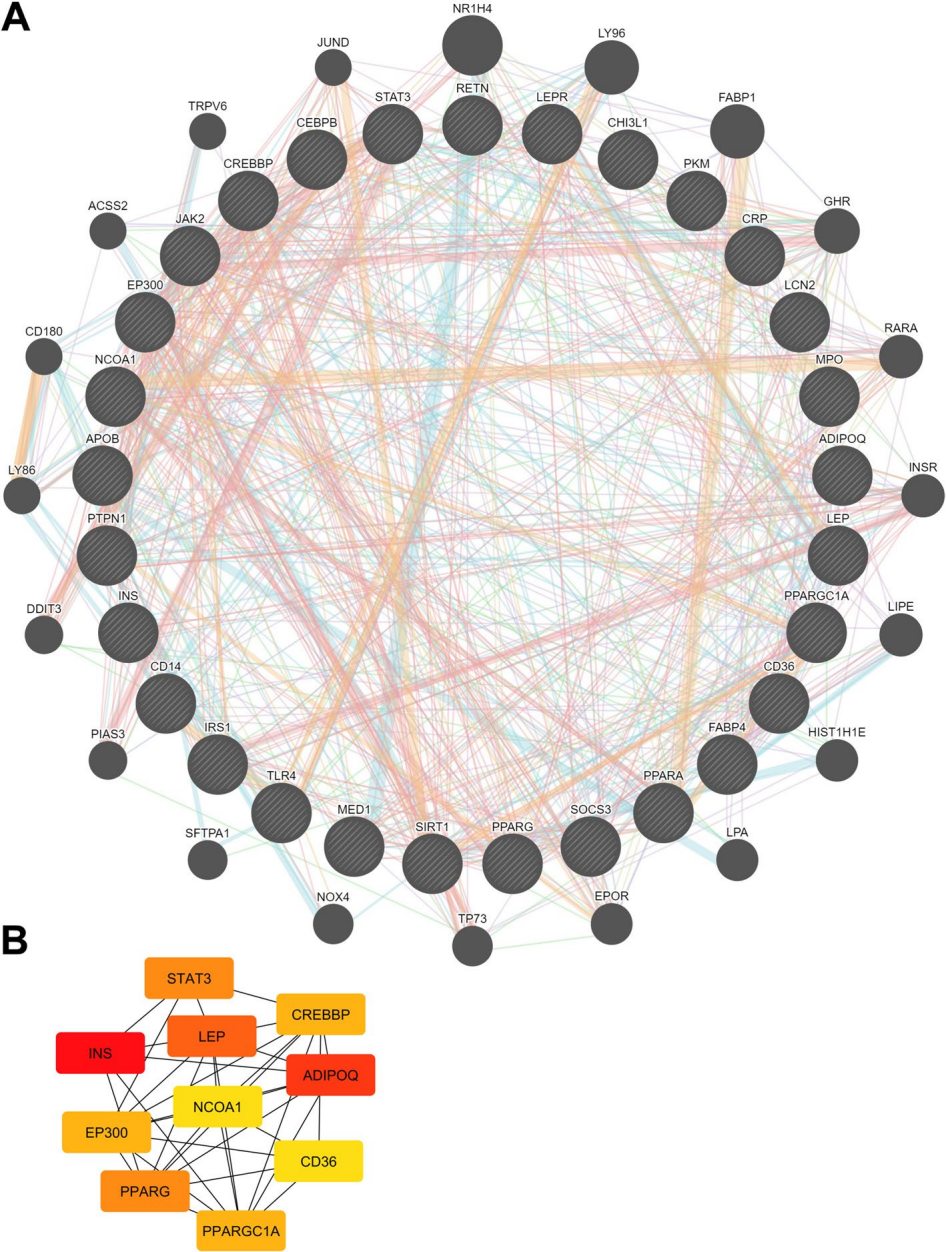
**A** PPI network of TR as analyzed using geneMANIA. **B** Top ten hub genes analyzed using the degree method of CytoHubba

PPI network analysis using STRING version 11.0 produced a network consisting of 29 nodes, 141 edges, an average node degree of 9.72, an average local clustering coefficient of 0.69, an expected edge number of 18, and a PPI enrichment *p*-value < 1.0e-16 (Fig. 3A). Hub gene selection based on degree score methods produced the top 10 proteins with the highest scores: INS, ADIPOQ, LEP, PPARG, STAT3, PPARGC1A, CREBBP, EP300, NCOA1, and CD36 (Fig. 3B, Table 1).

**Table 1:** Top 10 network string interactions ranked using the Degree method

No	Protein Symbol	Degree Score
1	INS	21
2	ADIPOQ	19
3	LEP	18
4	PPARG	14
5	STAT3	14
6	PPARGC1A	13
7	CREBBP	13
8	EP300	13
9	NCOA1	11
10	CD36	11

### Functional annotation of the TR

**Fig. 4:**
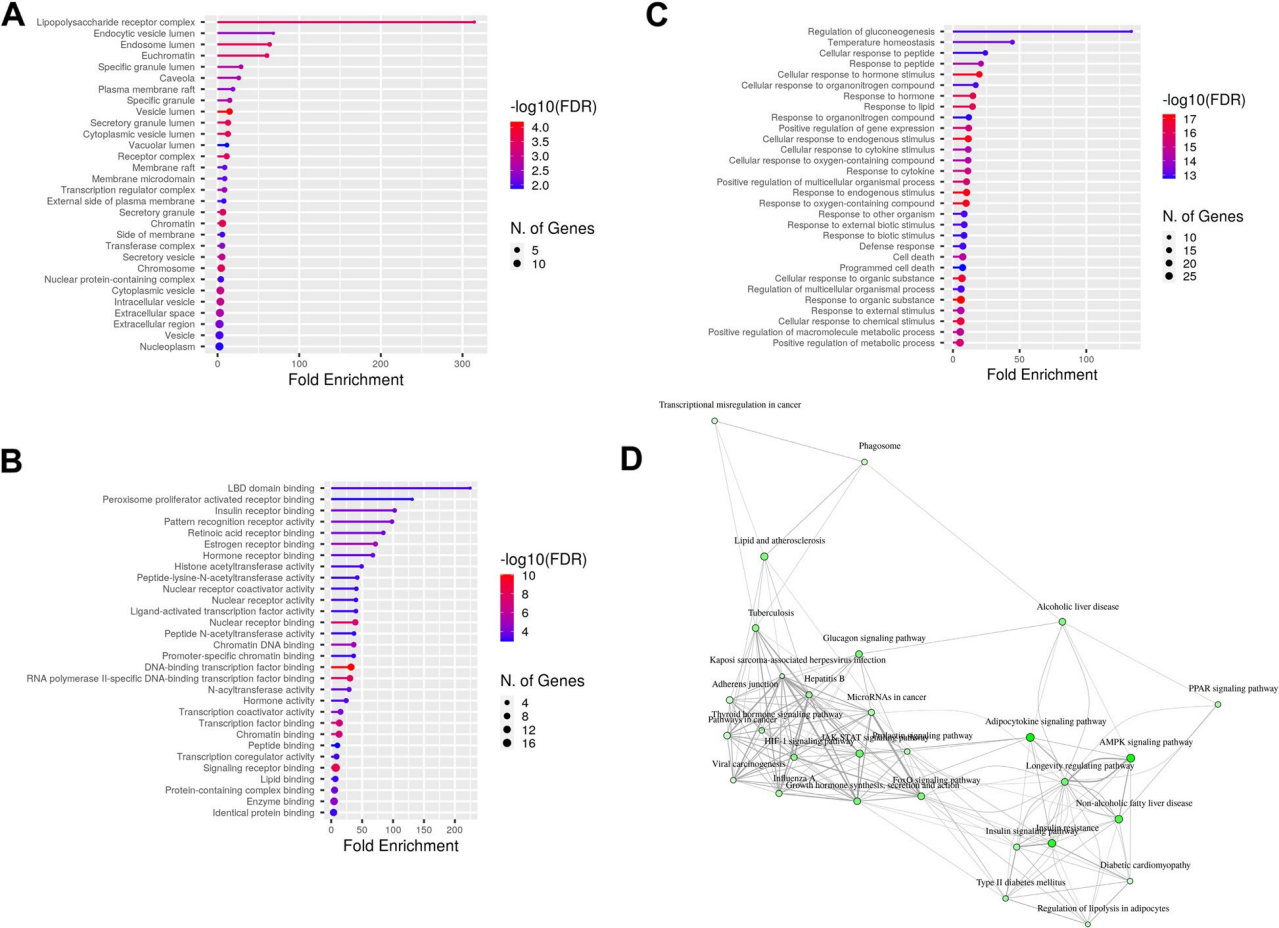
Functional annotation of the TR, including gene ontology enrichment analysis of **A** cellular components, **B** molecular functions, **C** biological processes, and **D** pathway enrichment network analysis. Fisher’s exact test was used in functional annotation of TR. *P*-value < 0.05 obtained using the Benjamini–Hochberg procedure was considered a threshold for significant value

Functional annotation analysis included gene ontology, consisting of biological processes, cellular components, and molecular functions. The TR is in several locations, including the lipopolysaccharide receptor complex, endosome lumen, and chromosome (Fig. 4A). TR plays a role in several molecular functions, including peroxisome proliferator-activated receptor and transcription factor binding (Fig. 4B). TR regulates critical biological processes, such as cellular responses to cytokine stimuli and lipids (Fig. 4C). Analysis of the pathway enrichment network analysis demonstrated that TR regulates adipocytokine, AMPK, and PPAR signaling pathways and miRNAs in cancer (Fig. 4D).

### Analysis of genetic alterations in selected TR

**Fig. 5:**
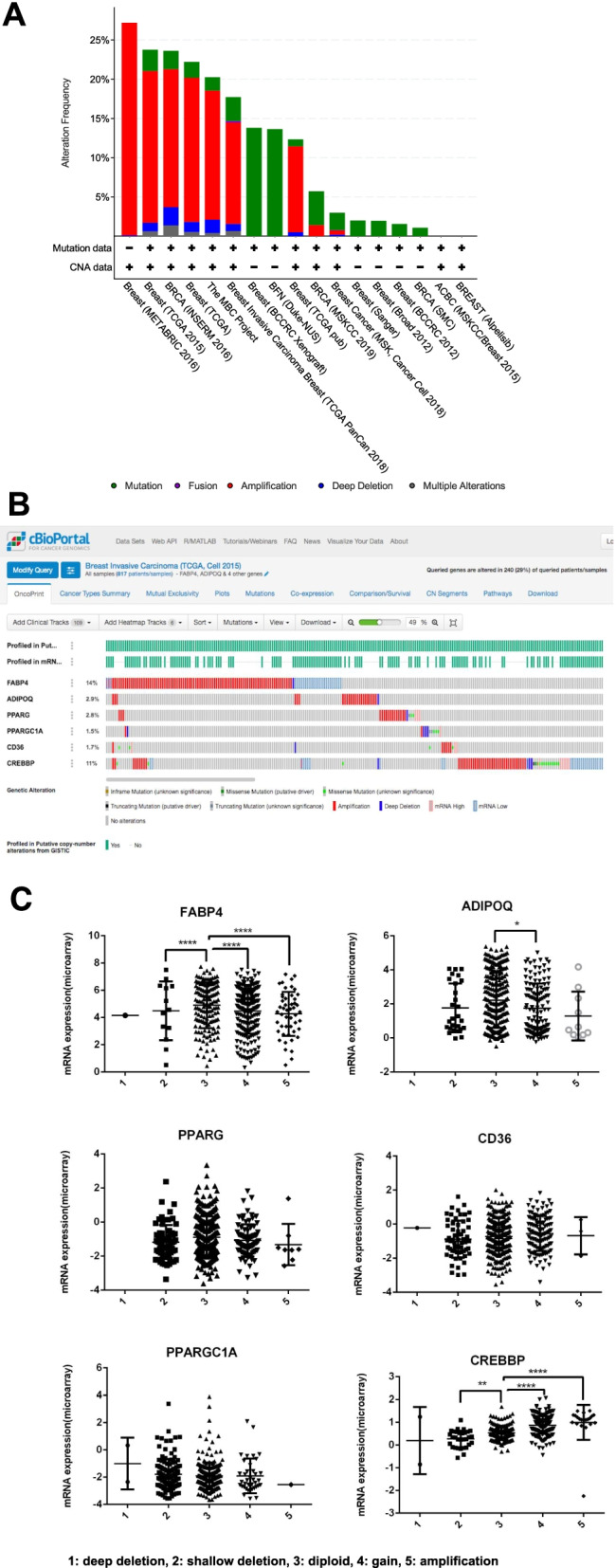
**A** Recaps of alterations in *FABP4, ADIPOQ, PPARG, PPARGC1A, CD36*, and *CREBBP* among breast cancer studies in the cBioportal database. **B** Oncoprint analysis showed genetic alterations of *FABP4, ADIPOQ, PPARG, PPARGC1A, CD36*, and *CREBBP* in breast cancer samples from the TCGA study by Ciriello et al. (2015). **C** Copy number alterations in *FABP4, ADIPOQ, PPARG, PPARGC1A, CD36*, and *CREBBP* in breast cancer samples from the TCGA study by Ciriello et al. (2015). Alterations included 1: deep deletion, 2: shallow deletion, 3: diploid, 4: gain, and 5: amplification. mRNA levels in each group were analyzed using one-way ANOVA with Tukey’s multiple comparisons test. Significances are shown as * for *p* < 0.05, ** for *p* < 0.01, and **** for *p* < 0.001

Genetic alterations in the selected TR were analyzed using cBioportal. *FABP4*, *ADIPOQ, PPARG, PPARGC1A, CD36*, and *CREBBP* were selected as query gene symbols and analyzed using cBioportal. *ADIPOQ, PPARG, FABP4,* and *PPARGC1* were selected based on the degree method using CytoHubba. *ADIPOQ, PPARG,* and *CD36* were the DTPs from RGZ. *ADIPOQ, PPARGC1A,* and *CD36* were DTPs involved in AMPK signaling. *PPARG, ADIPOQ, CD36*, and *FABP4* are involved in PPAR signaling. The TCGA study by Ciriello et al. [31] showed alterations in approximately 24% of the population (Fig. 5A) and was therefore choosen for further assesment. Oncoprint analysis revealed genetic alterations in *FABP4* (14%), *ADIPOQ* (2.9%), *PPARG* (2.8%), *PPARGC1A* (1.5%), *CD36* (1.7%), and *CREBBP* (11%) in patients with breast cancer in the TCGA study (Fig. 5B). Further mutual exclusivity analysis revealed that only one gene pair, *ADIPOQ-CD36*, co-occurred (Table 2).

**Table 2:** Mutual exclusivity analysis of target genes

A	B	Log2 Odds Ratio	*p*-Value	Tendency
*ADIPOQ*	*CD36*	> 3	0.017	Co-occurrence

The copy number alteration analysis showed that the mRNA level of *FABP4* was significantly lower in the shallow deletion and higher in the gain and amplification (Fig. 5C). The mRNA level of *ADIPOQ* was significantly higher in the gain condition. In addition, the mRNA level of *CREBBP *was significantly lower in the shallow deletion, and significantly higher in the gain and amplification.

### DNA methylation analysis of selected TR

We demonstrated a heatmap and prognostic value of DNA methylation clustering of the expression levels of *FABP4, ADIPOQ, PPARG, PPARGC1A, CD36*, and *CREBBP* in breast cancer (Supplementary Fig. 1). The highest levels of DNA methylation in patients with breast cancer were as follows: cg10062803 and cg14152613 of *FABP4*; cg06842886, cg14584085, and cg21978128 of *ADIPOQ*; cg07895576 and cg16827534 of *PPARG*; cg09427718, cg06772578, and cg08550435 of *PPARGC1A*; cg05345249 of *CD36*; cg16560077, cg01963870, cg27390443, cg27318635, cg03140190, and cg05898629 of *CREBBP*.

### Analysis of the gene expression in selected TR

**Fig. 6:**
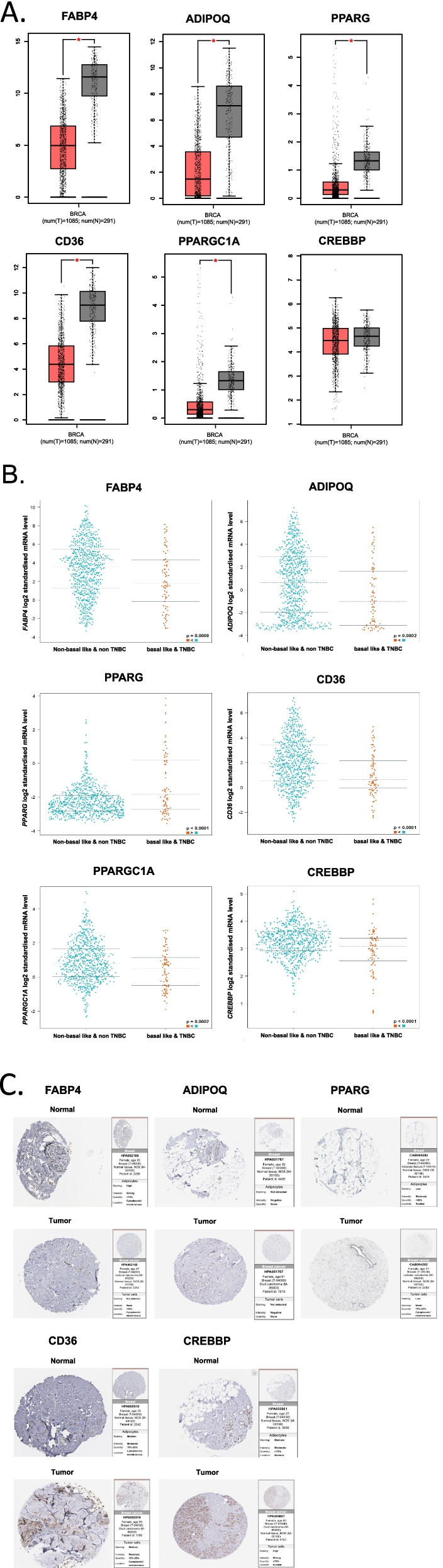
**A** mRNA levels of *FABP4, ADIPOQ, PPARG, PPARGC1A, CD36*, and *CREBBP* in breast cancer samples and adjacent normal breast tissues were analyzed using the GEPIA database. The method for differential analysis was one-way ANOVA. Statistical significance of differences in mRNA levels was set at *p* < 0.01 (*). **B** Analysis of gene expression of *FABP, ADIPOQ, PPARG, CD36, PPARGC1A*, and *CREBBP* in basal-like and TNBC cells with bc-GenExMiner using TCGA study data. The difference of gene expression in the different population groups was analyzed using Welch’s test. Statistical significance was set at *P*-value < 0.01. **C** Protein level of FABP4, ADIPOQ, PPARG, PPARGC1A, CD36, and CREBBP in normal and breast tumor tissues were analyzed using the Human Protein Atlas (HPA)

TR mRNA levels in breast cancer cells and adjacent tissues were checked using the GEPIA database. The mRNA expression levels of *FABP4, ADIPOQ, PPARG, CD36*, and *PPARGC1A* were significantly lower in patients with breast cancer (Fig. 6A), whereas the mRNA levels of *CREBBP *were not different between patients with breast cancer and normal breast tissues. Analysis of gene expression with bc-GenExMiner using TCGA data showed that the mRNA expression levels of *FABP, ADIPOQ, PPARG, CD36, PPARGC1A*, and *CREBBP* were significantly lower in basal-like and TNBC cells than in non-basal-like and TNBC cells (Fig. 6B).

### Protein expression in selected TR

Protein expression of FABP4 was not detected in normal breast tissue but was low in breast tumor tissues (Fig. 6C). Protein expression of ADIPOQ was not detected in normal breast or breast tumor tissues. Protein expression of PPARG was detected at low levels in both normal breast and breast tumor tissues. Protein expression of CD36 was detected at a low level in normal breast tissue and at a medium level in breast tumor tissue. PPARGC1A data was not available in the HPA database. Protein expression of CREBBP was detected at a medium level in both normal breast and breast tumor tissues. In general, the protein levels of TR were low, except for CREBBP, indicating the potential of RGZ treatment to inhibit angiogenesis by increasing the protein expression.

### Kaplan–Meier survival analysis

**Fig. 7:**
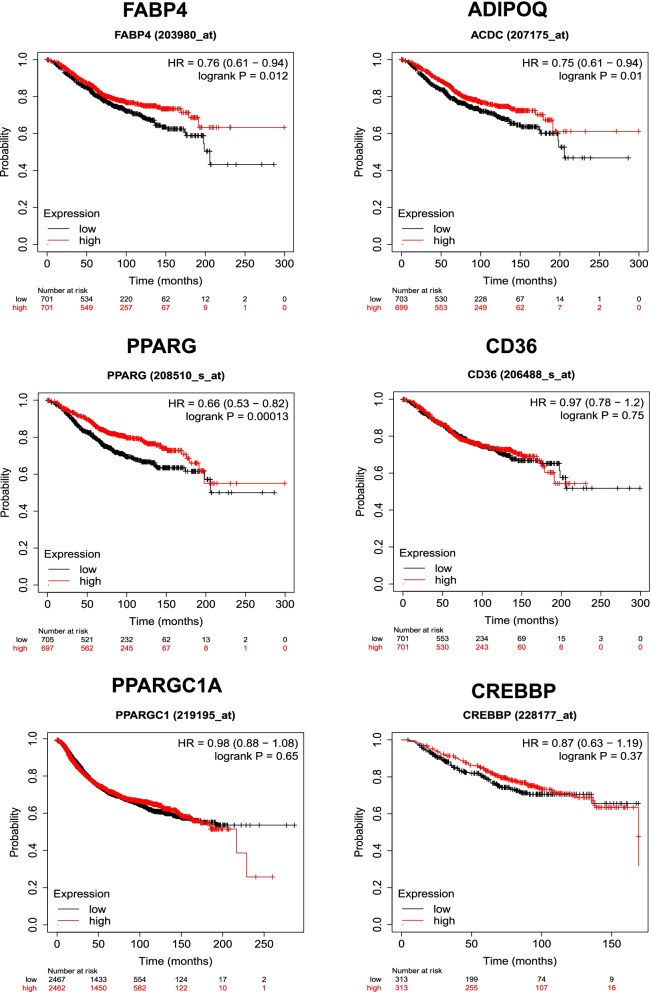
Overall survival in patients with breast cancer related to the mRNA levels of *FABP4, ADIPOQ, PPARG, PPARGC1A, CD36*, and *CREBBP*. The plot was considered significant if logrank was *p* < 0.05

The prognostic value of TR expression in breast cancer was analyzed using Kaplan–Meier survival rate based on OS. Patients with breast cancer who had low mRNA expression levels of *FABP4* (log-rank *P* = 0.012), *ADIPOQ* (log-rank P = 0.01), and *PPARG* (log-rank *P* = 0.00013) had worse OS than those with high mRNA levels (Fig. 7). Moreover, patients with breast cancer showed no significant difference in OS between low- and high-expressing cells of *CD36* (log-rank *P* = 0.75), *PPARGC1A* (log-rank *P* = 0.65), and *CREBBP* (log-rank *P* = 0.37). Additionally, expression levels of DNA methylation analyses revealed that cg14152613 and cg19422565 of *FABP4*; cg06842886 and cg16126291 of *ADIPOQ*; cg04632671, cg06573644, cg27095527, cg18537222, cg25929976, and cg16827534 of *PPARG*; cg11270806 and cg27461259 of *PPARGC1A*; cg26138637 and cg18508525 of *CD36*; and cg04818078 and cg05194552 of *CREBBP* had the highest levels of DNA methylation and strong predictive value in patients with breast cancer (Supplementary Table 4).

### Correlation analysis of immune cell infiltration with TR

Purity was negatively correlated with the expression of *FABP4* (Rho = − 0.24, *p* = 1.35e-03), *ADIPOQ* (Rho = − 0.296, *p* = 6.98e-05), *PPARG*, (Rho = − 0.211, *p* = 5.05e-03), and *CD36* (Rho = − 0.249, *p* = 9.10e-04) (Table 3, Supplementary Fig. 2). B-cell infiltration was negatively correlated with the expression level of *CD36* (Rho = − 0.162, *p* = 3.26e-02). CD8+ cells were positively correlated with *PPARGC1A* (Rho = 0.234, *P* = 1.9e− 03) and *CREBBP* (Rho = 0.2, *P* = 8.10e-03). CD4+ cell infiltration was positively correlated with *FABP4* (Rho = 0.251, *p* = 8.30e-04) and *ADIPOQ* (Rho = 0.264, *p* = 4.28e-04). Dendritic cell infiltration was positively correlated with *CREBBP* (Rho = 0.229, *p* = 2.39e-03). Cancer-associated fibroblast infiltration was positively correlated with the expression levels of *FABP4* (Rho = 0.283, *p* = 1.52e-04), *ADIPOQ* (Rho = 0.213, *p* = 4.74e-03), *PPARG* (Rho = 0.199, *p* = 8.56e- 03), *CD36* (Rho = 0.326, *p* = 1.12e-05), *PPARGC1A* (Rho = 0.198, *p* = 8.73e-03), and *CREBBP* (Rho = 0.186, *p* = 1.4e-02). Macrophage cell infiltration was positively correlated with the expression levels of *FABP4* (Rho = 0.174, *p* = 2.14e-02) and *CD36* (Rho = 0.246, *P* = 1.08e-03), whereas neutrophil cell infiltration was positively correlated with *CREBBP* (Rho = 0.19, *p* = 1.19e-02).

**Table 3:** Correlation between TR expression and Immune infiltration was analyzed using TIMER. Significant values are in bold

Description		*FABP4*	*ADIPOQ*	*PPARG*	*CD36*	*PPARGC1A*	*CREBBP*
Purity	Rho	−0.24	−0.296	−0.211	− 0.249	0.06	0.013
	p	**1.35e-03**	**6.98e-05**	**5.05e-03**	**9.10e-04**	4.29e-01	8.63e-01
B cell	Rho	−0.032	−0.059	−0.108	− 0.162	−0.038	0.009
	p	6.72e-01	4.38e-01	1.56e-01	**3.26e-02**	6-23e-01	9.10e-01
CD8+	Rho	0	0.015	0.054	0.085	0.234	0.2
	p	9.96e-01	8.74e-01	4.8e-01	2.65e-01	**1.9e-03**	**8.10e-03**
CD4+	Rho	0.251	0.264	0.027	0.128	0.038	0.138
	p	**8.30e-04**	**4.28e-04**	7.2e-01	9.34e-02	6.14e-01	6.87e-02
Dendritic cells	Rho	0.111	0.09	−0.011	0.068	0.059	0.229
	p	1.46e-01	2.38e-01	8.88e-01	3.72e-01	4.39e-01	**2.39e-03**
Cancer-associated fibroblasts	Rho	0.283	0.213	0.199	0.326	0.198	0.186
	p	**1.52e-04**	**4.74e-03**	**8.56e-03**	**1.12e-05**	**8.73e-03**	**1.4e-02**
Macrophage	Rho	0.174	0.129	0.025	0.246	−0.145	−0.095
	p	**2.14e-02**	9.07e-02	7.47e-01	**1.08e-03**	5.62e-02	2.21e-01
Neutrophils	Rho	−0.026	0.023	0.031	0.046	−0.095	0.19
	p	7.34e-01	7.63e-01	6.87e-01	5.43e-01	2.14e-01	**1.19e-02**

## Discussion

This study analyzed the potential of RGZ as an anticancer drug using bioinformatics approaches. We identified 29 protein targets that could be potential RGZ targets for inhibiting breast cancer angiogenesis (TR). Oncoprint analysis revealed genetic alterations in *FABP4* (14%), *ADIPOQ* (2.9%), *PPARG* (2.8%), *PPARGC1A* (1.5%), *CD36* (1.7%), and *CREBBP* (11%) in patients with breast cancer in a TCGA study. DNA methylation is an epigenetic alteration that is involved in breast cancer progression [32]. Methylation of the CpG island gene is known to predict breast cancer progression [33]. DNA methylation analysis revealed that the predictive significance of *FABP4, ADIPOQ, PPARG, PPARGC1A, CD36*, and *CREBBP* in a specific CpG was significant in the emergence of breast cancer. This phenomenon indicates the importance of TR as a therapeutic target for breast cancer angiogenesis.

*ADIPOQ* encodes adiponectin, which is expressed only in adipose tissues [34]. Mutations in this gene result in adiponectin deficiency. Adiponectin levels are regulated by PPARγ signaling through transcriptional and post-transcriptional mechanisms [35]. Adiponectin is secreted by adipose tissue and exhibits anticancer, anti-inflammatory, and antioxidant activities [36]. A recent study showed that obesity is a risk factor that is strongly associated with postmenopausal breast cancer [37]. A meta-analysis showed that the genetic variation in *ADIPOQ* named T45G, is not related to insulin resistance or blood glucose [38]. Polymorphisms in *ADIPOQ* affect serum adiponectin levels and are associated with breast cancer risk. For example, a previous study found a decrease in serum adiponectin levels and an increase in the risk of breast cancer in patients in Mexico [39]. Genetic variation in *ADIPOQ*, rs1501299 (G267T), decreases serum adiponectin levels in patients with breast cancer, and an association between *ADIPOQ *genetic variation and breast cancer risk has been found in patients with postmenopausal breast cancer in Egypt [40]. A recent study found that *ADIPOQ* is negatively regulated by miR-9-5p, which plays a role in the sensitivity of breast cancer cells to tamoxifen [41]. The effect of RGZ on ADIPOQ on angiogenesis in breast cancer is an interesting topic worth exploring.

*PPARG* encodes PPARγ. Peroxisome proliferator-activated receptor forms heterodimers with other receptors such as retinoic acid receptors [42]. PPARγ plays an important role in metabolic reprogramming and oxidative phosphorylation, such as electron transport and activation of reactive oxygen species (ROS)-metabolizing enzymes [43]. PPAR signaling has implications in the pathophysiology of skeletal muscle dysfunction in patients with breast cancer [44]. RGZ activates PPARg signaling in endothelial cells [45]. RGZ inhibits metastasis and migration, decreases MMP-2 expression, and prevents angiogenesis by blocking the vascular endothelial growth factor (VEGF) pathway in SGC-7901 gastric cancer cells [46]. In addition, RGZ reduces the risk of breast cancer in patients with T2DM in Taiwan [47]. *PPARGC1A* encodes peroxisome proliferator-activated receptor G coactivator-1a (PGC-1a), a transcriptional coactivator of nuclear receptors and a subfamily member of PPARg [48]. A previous study showed that PGC-1a is a key regulator of angiogenesis and lipid and carbohydrate metabolism [49, 50]. Therefore, further investigation of RGZ-PPARγ signaling in breast cancer angiogenesis is warranted.

CD36 is a cellular scavenger that mediates lipid uptake, recognition of immune responses, inflammation, and apoptosis [51]. CD36 is an 88 KDa transmembrane glycoprotein receptor expressed in various cells, such as monocytes, macrophages, endothelial cells, and adipose cells [52]. CD36 prevents angiogenesis by binding to thrombospondin-1, promoting apoptosis, and inhibiting the VEGFR2 pathway in the endothelial microvessels [53]. In gastric cancer cells, phosphatidylinositol transfer upregulates PPARG and CD36 [53]. RGZ increased the expression of CD36 in rat muscle cells [54]. The effect of RGZ on CD36 in breast cancer angiogenesis is a strategic approach for drug development.

*FABP4* or the gene encoding for fatty acid-binding protein 4 (FABP4) is also known as adipocyte FAB or aPA2 and is expressed by adipocytes and macrophages [55]. FABP4 is a chaperone protein found in the cytoplasm, is expressed in adipocytes and myeloid cells, and plays a role in the ubiquitination and degradation of PPARG proteosomes [56]. Several studies have shown that FABP4 plays a role in carcinogenesis. FABP4 is found in stromal cells and can trigger cancer growth by supplying energy to cancer cells or increasing angiogenesis in ovarian cancer cells [57]. Harjes investigated the role of FABP4 and found that *FABP4 *knockdown inhibited growth, metastasis, and angiogenesis of ovarian cancer in vitro and in vivo [58]. FABP4 suppresses the proliferation and invasion of hepatocellular carcinoma cells and is a predictor of poor prognosis [59]. One study revealed that FABP4 is a pivotal regulator of metastasis in ovarian cancer cells through miR-409-3p modulation [60]. In addition, PPARG signaling activation causes lipolysis mediated by FABP4 and inhibits lung and renal cancer cell growth [61]. Another study showed that serum FABP4 levels increased in patients with colorectal cancer in China compared with normal test subjects, indicating that FABP4 is a risk factor and a potential biomarker [62]. A recent study showed that FABP4 triggers invasion and metastasis in colon cancer through the regulation of fatty acid transport [63]. This study also revealed that *FABP4 *overexpression triggers epithelial–mesenchymal transition (EMT), upregulates Snail, MMP-2, and MMP-9, and decreases E-cadherin expression. Taken together, these studies indicate that FABP4 is a potential target of RGZ in angiogenesis, and further comprehensive studies are warranted to explore the molecular mechanism of RGZ-targeting FABP4.

*CREBBP* encodes cyclic AMP-responsive element-binding protein (CREB)-binding protein or CBP, a protein involved in the pathological regulation of diseases such as schizophrenia, embryonic development, and growth control [64]. CREBBP or CBP stabilizes transcription complexes but also exerts intrinsic histone acetyl transferase (HAT) activity in chromatin remodeling [65]. Mutations in *CREBBP *have been found in patients with Rubinstein Taybi syndrome and acute lymphoid leukemia [66]. Previous studies have shown that CREBBP plays a role in cancer progression. Deletion of *CREBBP* occurs in 18.3% of patients with acute lymphoblastic leukemia and encodes a transcriptional coactivator and HAT from CREBBP [66]. Genetic polymorphisms and transcriptional regulation of the *CREBBP *gene have been observed in patients with large B-cell lymphoma. However, the difference in mRNA levels was not statistically significant between low and high levels of OS and progression-free survival [67]. *CREBBP* expression abnormalities have been found in patients with lung [68] and prostate [69] cancer [69]. Wang demonstrated that *CREBBP* mRNA levels are correlated with the expression of metastasis regulator genes such as catenin, cadherin, and EGFR [68]. Further studies on RGZ activity targeting CREBBP in breast cancer angiogenesis are required.

KEGG pathway enrichment analysis demonstrated that TR regulated adipocytokine, AMPK, PPAR, TLR4, and hypoxia-inducible factor (HIF) signaling pathways. Adipocytokines are polypeptides produced by adipocytes that play a role in signaling and are responsible for the development of breast cancer [70]. Activation of HIF signaling increases the expression of VEGF, glycolysis, angiogenesis, and apoptosis regulatory genes [71]. Activation of PPARγ signaling modulated the formation of ROS and the activation of NF-κB and HIFα signaling in mice with an allergic respiratory tract [72]. Moreover, HIF signaling plays an important role in angiogenesis and breast cancer development; thus, HIFs are important therapeutic targets [73].

RGZ targets adiponectin and HIF signaling pathways It increases serum leptin levels in patients with T2DM [74]. Yee et al. conducted a short clinical trial in patients with breast cancer and found that RGZ treatment increased serum adiponectin levels without serious side effects [9]. Li et al. showed that RGZ attenuated the decrease in *ADIPOQ* mRNA expression in adipose tissues [75]. Another study showed that activation of PPAR signaling by RGZ attenuates HIF signaling [76].

A previous study showed that Toll-like receptor 4 triggers angiogenesis in pancreatic cancer cells by regulating PI3K/Akt signaling [77]. The same authors also showed that TLR4 triggers angiogenesis by activating PI3K/Akt signaling, thereby inducing VEGF expression in pancreatic cancer cells. In esophageal cancer cells, PPARG signaling activation inhibited proliferation and induced apoptosis by inhibiting TLR4-dependent MAPK signaling [78]. Previous studies have revealed that RGZ inhibits TLR4 signaling. In addition, RGZ inhibits the release of TNF**α** induced by TLR4 signaling through the phosphorylation of p38, JNK, and MAPK during neuroinflammation [79]. A previous in vivo study revealed that RGZ attenuates apoptosis by inhibiting the TLR4/NF-κB signaling pathway in acute myocardial infarction [80]. However, the effects of RGZ on angiogenesis inhibition in breast cancer cells require further investigation.

Activated protein kinase (AMPK) signaling plays a role in regulating energy balance and cellular nutrition and indirectly inhibits p70S6 kinase, thereby preventing cell migration [81]. Several studies have demonstrated the importance of the AMPK signaling pathway in breast cancer development. Activation of AMPK signaling inhibits the growth of DU145 and PC3 prostate cancer cells by suppressing mTOR/p70S6K [82]. PPARγ transcriptional activity is inhibited by activated AMPK in hepatoma cells [83]. Activation of AMPK1 also triggers VEGF-induced angiogenesis [84]. AMPK plays an important role in chemoresistance and survival and is a potential therapeutic target for TNBC [85]. AMPK activation plays an important role in breast cancer development in postmenopausal women. RGZ suppresses the growth of lung cancer cells by upregulating the AMPK signaling-dependent pathway and downregulating the Akt/mTOR/p70S6K pathway [86]. RGZ inhibits PPARG and AMPK signaling in human nasopharyngeal cancer cells [87]. However, the mechanism of RGZ in breast cancer angiogenesis that targets PPARγ, HIF, TLR4, and AMPK signaling pathways needs to be clarified.

Analysis of the prognostic value related to TR expression showed that patients with breast cancer with low mRNA expression levels of *FABP4* (log-rank *P* = 0.012), *ADIPOQ* (log-rank P = 0.01), *PPARG* (log-rank *P* = 0.00013), and *PPARGC1A* (log-rank *P* = 0.02) had worse OS than those with high mRNA levels. Therefore, upregulation of TR during RGZ treatment increases the OS of patients with breast cancer. The analysis performed using TIMER 2.0 showed that B-cell infiltration was negatively correlated with CD36, which is expressed in B-cell subsets because of the immune response to antigens [88]. CD8 infiltration was negatively correlated with *PPARGC1A *and *CREBBP*. PGC-1α-overexpressing CD8+ T cells showed enhanced antitumor immunity in a mouse melanoma model [89].

CAF infiltration was positively correlated with *FABP4, ADIPOQ, PPARG, CD36, PPARGC1A*, and *CREBBP*. Macrophage infiltration was positively correlated with *FABP4* and *CD36* levels, whereas neutrophils were positively correlated with *CREBBP*. *FABP4* expression in macrophages is induced by activation of PPARγ signaling [90]. Phagocytosis, mediated by CD36 in apoptotic cells, plays an important role in fibrosis [91]. In addition, CD36 functions in tumor-associated immune cells, causing tumor intolerance and progression; thus, it has become a strategic target for cancer therapy [53]. CD36 is expressed in tumor cells, and CD36 deficiency is characterized by stromal tumor and high cancer risk [92]; the lower the CD36 stromal level, the more aggressive the tumor. Taken together, the correlation analysis of immune infiltration of TR emphasized the potential RGZ target gene against angiogenesis in breast cancer by regulating the immune response.

TR plays different roles in the progression of different subtypes of breast cancer. A study by Kim showed that only a few patients with breast cancer express FABP4, including luminal A (0.8%), luminal B (0.7%), HER2+ (6%), and TNBC (4%) [93]. Moreover, FABP4 levels significantly correlated with ER status in patients with breast cancer. FABP4 increases breast cancer cell proliferation in MCF-7 (luminal breast cancer) and MDA-MB-231 triple-negative breast cancer cells, but activation of fatty acid transporters only occurs in MCF-7 luminal breast cancer cells [94]. A previous study showed no correlation between clinicopathologic parameters, including ER, PR, and HER2 status, and FABP expression [95]. FABP4 also plays a critical role in the metastasis and stromal interaction of MDA-MB 231, triple-negative breast cancer cells (TNBC) [96]. Taken together, FABP4 expression levels were not different in any subtype of breast cancer but played a critical role in the progression of ER+ and TNBC.

A previous study demonstrated that serum [97] and protein levels of ADIPOQ were not significantly associated with breast tumor clinicopathology [98]. Recent studies have shown that ADIPOQ is a promising biomarker for TNBC [99] and that lower levels of ADIPOQ are associated with TNBC progression [100]. HER2 overexpression leads to upregulation of CD36 and FABP4 [101]. CD36 is highly expressed in TNBC and plays a role in the fatty acids uptake [102, 103]. Another study showed that CD36 was highly expressed in ER+, moderately expressed in HER2+, and low in TNBC [104]. CD36 increases proliferation and migration of ER+ breast cancer cells [104].

Interaction of ERα and PPARγ inhibits PI3K downstream signaling, which leads to the inhibition of MCF-7 ER+ cells [105]. Crosstalk between PPARG and ER suppresses the proliferation and migration of thyroid cancer cells [106]. In contrast, stimulation of PPARγ signaling leads to ER inhibition and induces apoptosis in papillary thyroid cancer cells [107]. Overexpression of HER2 induces upregulation of PPARG transcription and translation in ER+ MCF-7 cells [108]. Moreover, inhibition of PPARγ signaling by its antagonist inhibits breast cancer stem cells in the HER2+ subtype [109]. In contrast, stimulation of PPARγ signaling by PPAR agonists hampers the migration and metastasis of TNBC cells [110].

The expression of PGC-1α, encoded by *PPARGC1A*, is controlled by the β-catenin pathway in ER+ breast cancer cells [111]. A previous study showed that PGC-1α levels were higher in the HER2+ and the basal subtypes than in other subtypes, which also showed poor prognosis in both subtypes [112]. *CREBBP* amplification occurs in ER+ and TNBC but not in HER2+ subtypes [113]. Recently, CREBBP was identified as a novel driver of TNBC progression [114]. Taken together, modulation of PPARγ signaling and CREBBP depends on the breast cancer subtype.

**Fig. 8:**
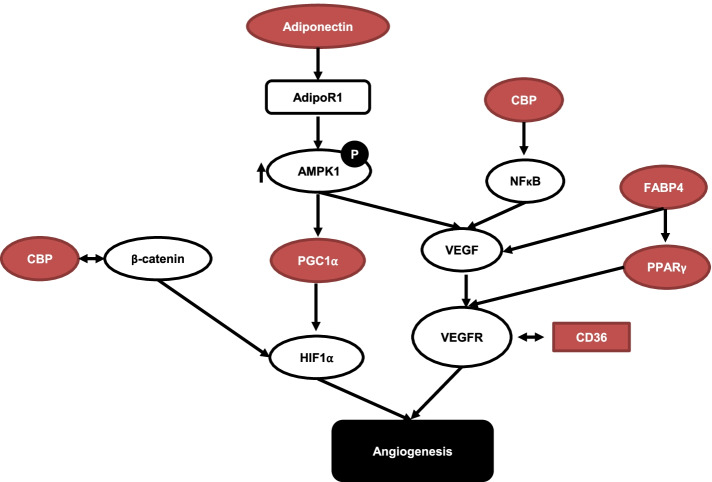
Proposed mechanism of RGZ for the inhibition of angiogenesis. The maroon shape indicates TR

This study highlighted six potential target genes that regulate angiogenesis. We propose a mechanism by which RGZ inhibits angiogenesis by targeting TR (Fig. 8). The binding of adiponectin to its receptor ADIPOR1 stimulates AMPK signaling and subsequently increases VEGF expression [115]. In skeletal muscle cells, the activation of AMPK signaling also increases VEGF mediated by PGC1α [116]. Activation of PGC-1α also increased the expression of hypoxia-inducible genes, including HIF-1α [117]. CBP increased the transactivation of NF-κB and its target genes, including VEGF, in endothelial progenitor cells [118]. PPARγ stimulates the expression of VEGFR2 and promotes angiogenesis in endothelial cells [119]. Fatty acids stimulate the expression of VEGF and FABP, which directly modulate angiogenesis in first-trimester placental trophoblast cells and FABP4 increases VEGF expression and induces angiogenesis [120]. Chu showed that CD36 forms a complex with VEGFR2 and promotes VEGF signaling, tube formation, and angiogenesis in microvascular endothelial cells [121]. Another recent study showed that the interaction between CBP and β-catenin increased HIF1a and angiogenesis; however, using a compound, such as E7386, to inhibit this interaction reversed the angiogenesis mechanism [122]. The results of the present study were obtained using a bioinformatics approach. Data mining using another database such as CMap, which connects drugs and gene experience profiles with a certain disease status and predicts the mechanism of the drugs in dealing with certain diseases, can be performed in the future. Further in vitro*,* in vivo, and clinical trials are needed to validate and develop RGZ as an antiangiogenic agent against breast cancer cells.

## Conclusion

In this study, the potential of RGZ as an antiangiogenic drug for breast cancer treatment was investigated. This study explored the potential of RGZ as an antiangiogenic agent in breast cancer therapy. We identified FABP4, ADIPOQ, PPARG, PPARGC1A, CD36, and CREBBP as potential targets of RGZ. We also investigated the potential role of TR as an immunotherapy target for RGZ in preventing breast cancer angiogenesis. Future study using in vitro and in vivo experiments are required to expand the therapeutic potential of RGZ against angiogenesis in breast cancer cells.

## Supplementary Information

**Additional file 1: Supplementary Table 1.** Direct target proteins (DTPs) and indirect target proteins (ITPs) of RGZ were analyzed using STITCH and STRING. **Supplementary Table 2.** Breast cancer angiogenesis regulatory genes. **Supplementary Table 3.** Targets of RGZ against breast cancer (BC) angiogenesis. **Supplementary Table 4.** MethSurv prognostic value of a single CpG from the *FABP4*, *ADIPOQ*, *PPARG*, *PPARGC1A*, *CD36*, and *CREBBP* in breast cancer.

**Additional file 2: Supplementary Fig. 1.** Heatmap of *FABP4*, *ADIPOQ*, *PPARG*, *PPARGC1A*, *CD36*, and *CREBBP* DNA methylation expression levels in breast cancer cells using MethSurv database. **Supplementary Fig. 2.** The correlation between TR and the level of immune cell infiltration was analyzed using TIMER 2.0.

## Data Availability

The data generated during and/or analysed during the current study are available on the supplementary files.

## Abbreviations

AMPK: Activated protein kinase
CREBBP: Cyclic AMP responsive element binding protein-binding protein
DTPs: Direct target proteins
FABP4: Fatty acid-binding protein 4
HAT: Histone acetyl transferase
HIFs: Hypoxia-inducible factors
ITPs: Indirect target proteins
OS: Overall survival
PPARγ: Peroxisome proliferator-activated receptor-gamma
PPARGC1A: Peroxisome proliferator-activated receptor G coactivator-1a
PPI: Protein–protein interaction
RGZ: Rosiglitazone
T2DM: Type 2 diabetes mellitus
TNBC: Triple-negative breast cancer
TR: Potential RGZ targets in inhibiting breast cancer angiogenesis
LR: Likelihood ratio
