# Supplementary material for: Bioinformatics analysis reveals the potential target of rosiglitazone as an antiangiogenic agent for breast cancer therapy
Source: BMC Genom Data. 2022 Sep 16;23:72. doi: 10.1186/s12863-022-01086-2 (PMC9482259; doi:10.1186/s12863-022-01086-2)
Supplement: Supplementary file 2 — Additional file 2: Supplementary Fig. 1. Heatmap of FABP4, ADIPOQ, PPARG, PPARGC1A, CD36, and CREBBP DNA methylation expression levels in breast cancer cells using MethSurv database. Supplementary Fig. 2. The correlation between TR and the level of immune cell infiltration was analyzed using TIMER 2.0. [file 12863_2022_1086_MOESM2_ESM.pptx]

## Slide 1
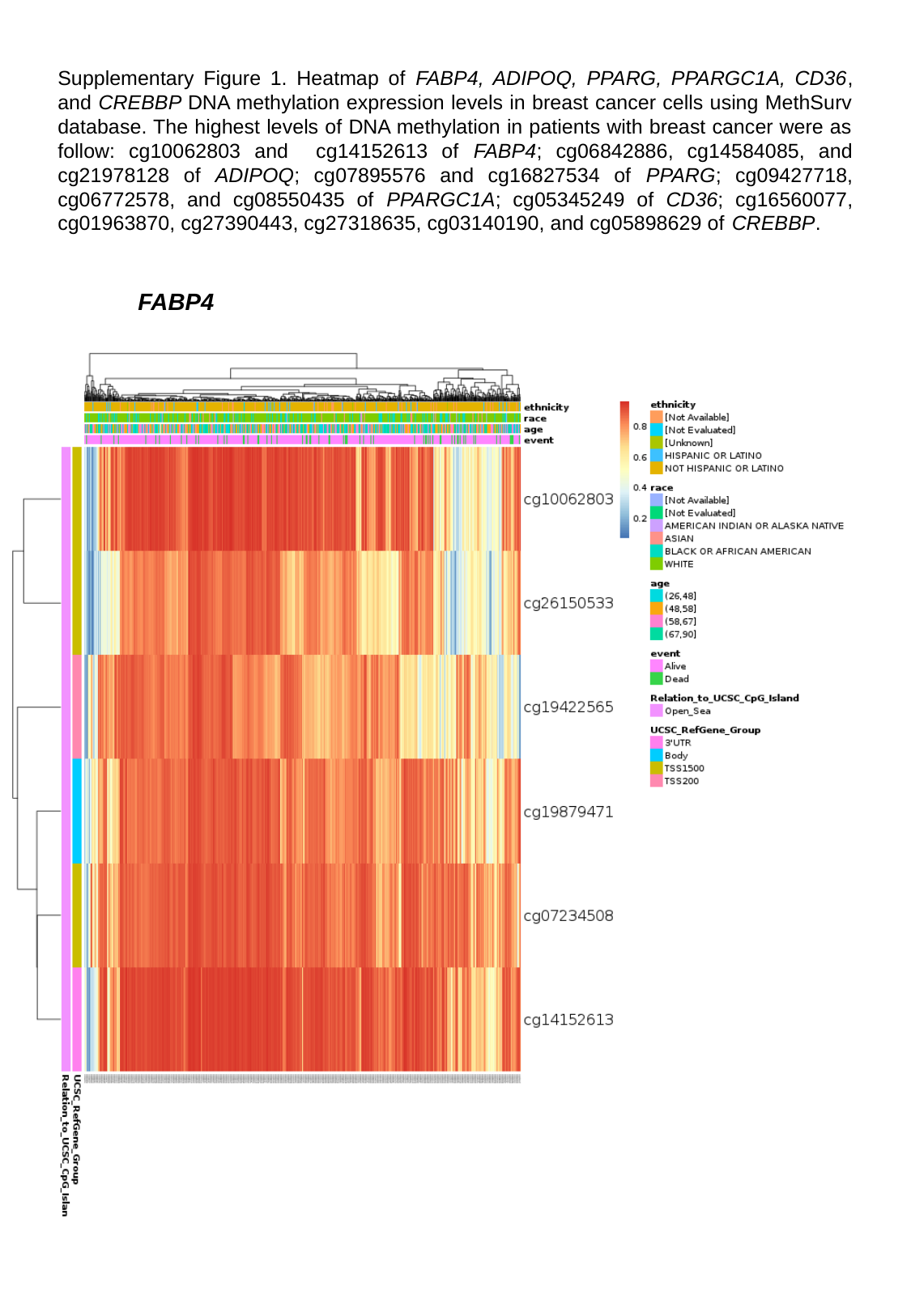

# Supplementary Figure 1. Heatmap of FABP4, ADIPOQ, PPARG, PPARGC1A, CD36, and CREBBP DNA methylation expression levels in breast cancer cells using MethSurv database. The highest levels of DNA methylation in patients with breast cancer were as follow: cg10062803 and cg14152613 of FABP4; cg06842886, cg14584085, and cg21978128 of ADIPOQ; cg07895576 and cg16827534 of PPARG; cg09427718, cg06772578, and cg08550435 of PPARGC1A; cg05345249 of CD36; cg16560077, cg01963870, cg27390443, cg27318635, cg03140190, and cg05898629 of CREBBP.
FABP4

## Slide 2
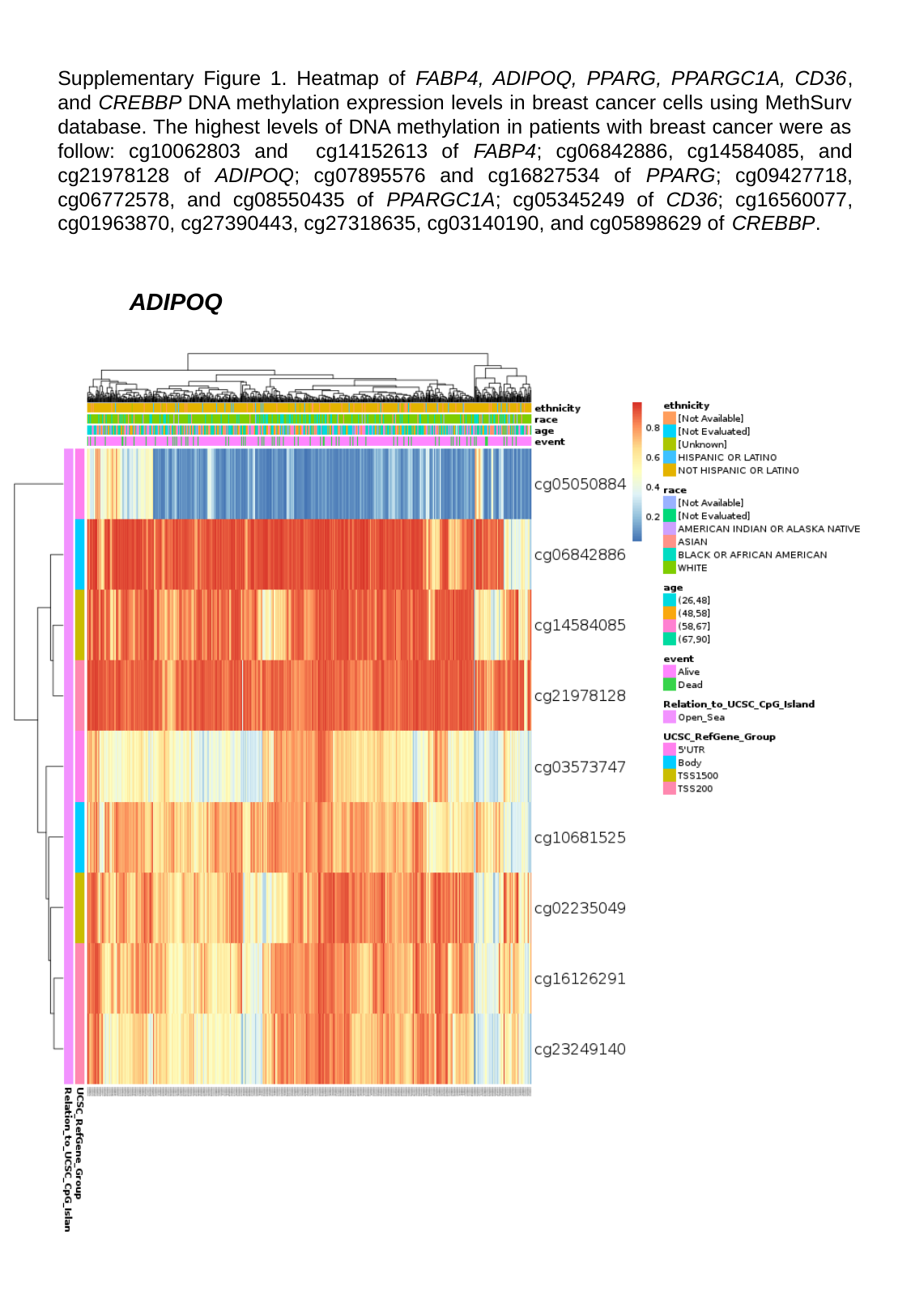

# Supplementary Figure 1. Heatmap of FABP4, ADIPOQ, PPARG, PPARGC1A, CD36, and CREBBP DNA methylation expression levels in breast cancer cells using MethSurv database. The highest levels of DNA methylation in patients with breast cancer were as follow: cg10062803 and cg14152613 of FABP4; cg06842886, cg14584085, and cg21978128 of ADIPOQ; cg07895576 and cg16827534 of PPARG; cg09427718, cg06772578, and cg08550435 of PPARGC1A; cg05345249 of CD36; cg16560077, cg01963870, cg27390443, cg27318635, cg03140190, and cg05898629 of CREBBP.
ADIPOQ

## Slide 3
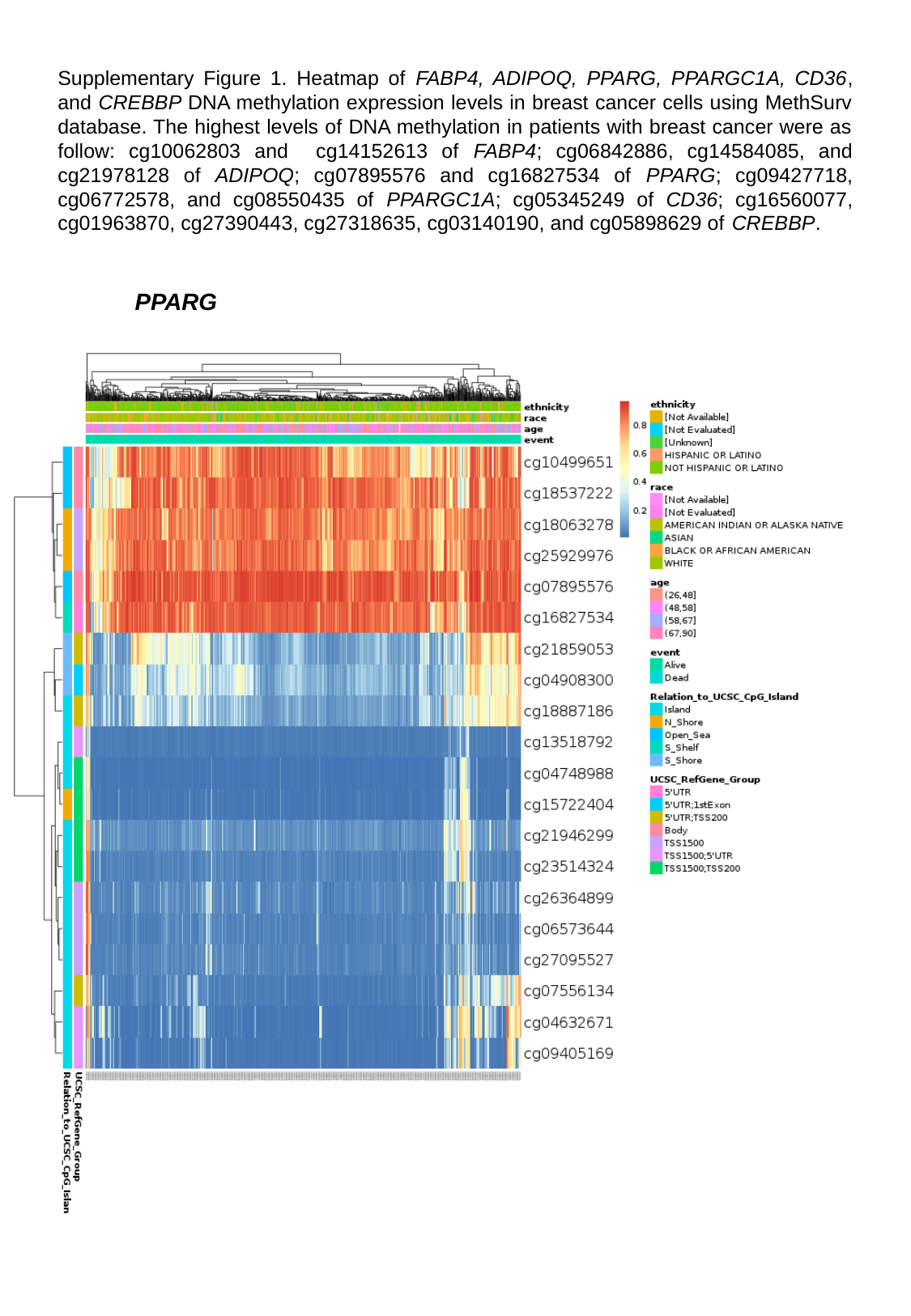

# Supplementary Figure 1. Heatmap of FABP4, ADIPOQ, PPARG, PPARGC1A, CD36, and CREBBP DNA methylation expression levels in breast cancer cells using MethSurv database. The highest levels of DNA methylation in patients with breast cancer were as follow: cg10062803 and cg14152613 of FABP4; cg06842886, cg14584085, and cg21978128 of ADIPOQ; cg07895576 and cg16827534 of PPARG; cg09427718, cg06772578, and cg08550435 of PPARGC1A; cg05345249 of CD36; cg16560077, cg01963870, cg27390443, cg27318635, cg03140190, and cg05898629 of CREBBP.
PPARG

## Slide 4
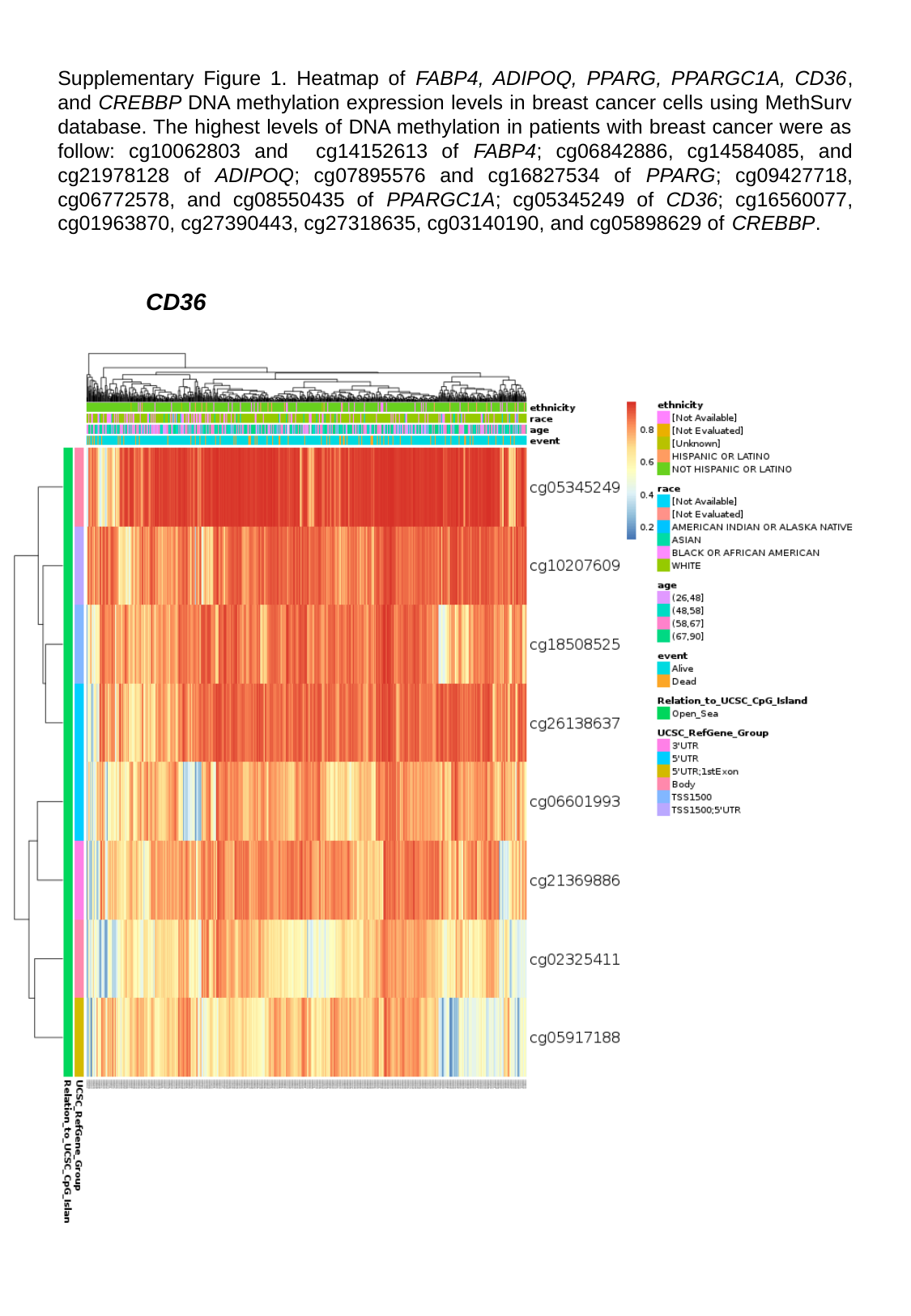

# Supplementary Figure 1. Heatmap of FABP4, ADIPOQ, PPARG, PPARGC1A, CD36, and CREBBP DNA methylation expression levels in breast cancer cells using MethSurv database. The highest levels of DNA methylation in patients with breast cancer were as follow: cg10062803 and cg14152613 of FABP4; cg06842886, cg14584085, and cg21978128 of ADIPOQ; cg07895576 and cg16827534 of PPARG; cg09427718, cg06772578, and cg08550435 of PPARGC1A; cg05345249 of CD36; cg16560077, cg01963870, cg27390443, cg27318635, cg03140190, and cg05898629 of CREBBP.
CD36

## Slide 5
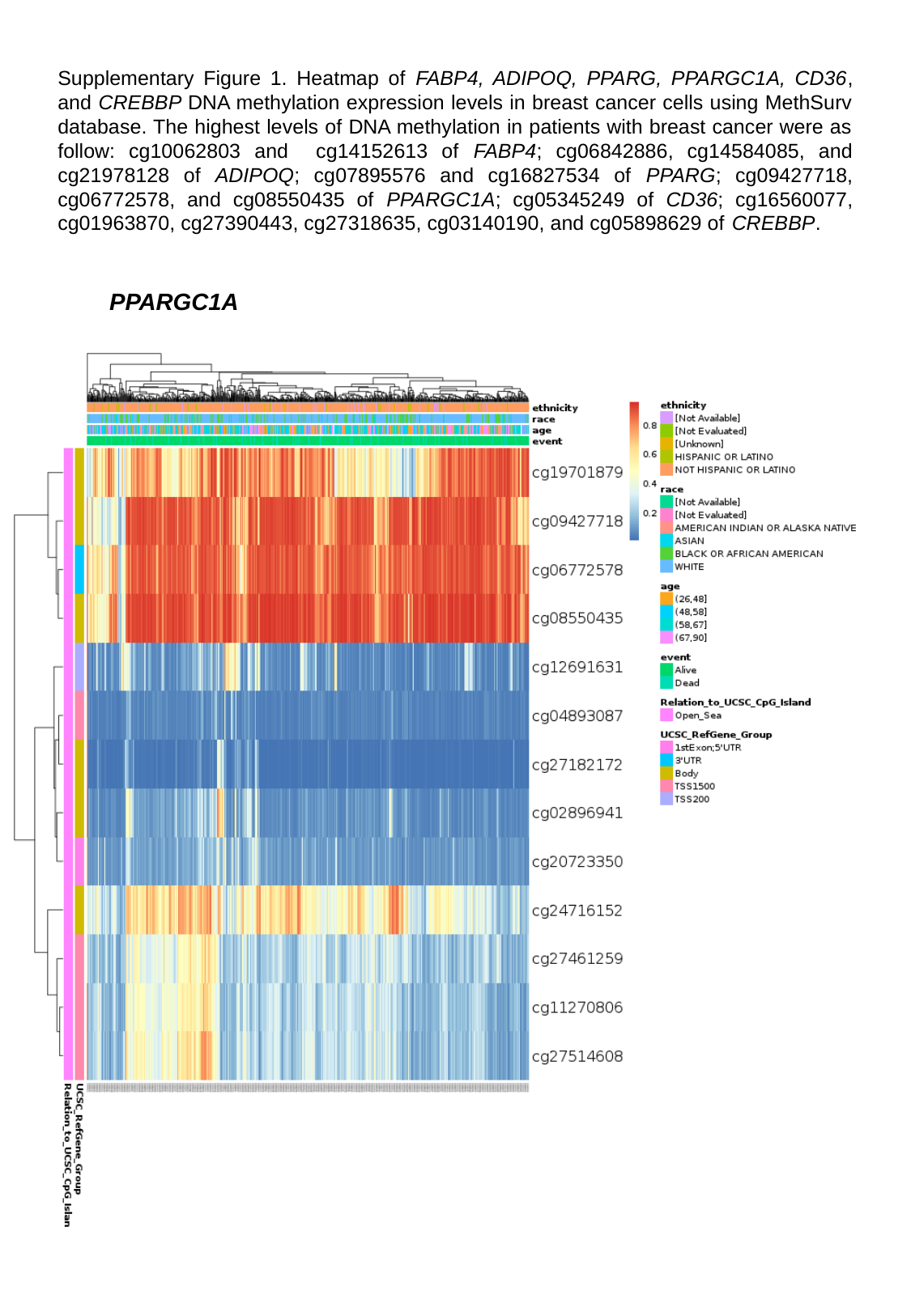

# Supplementary Figure 1. Heatmap of FABP4, ADIPOQ, PPARG, PPARGC1A, CD36, and CREBBP DNA methylation expression levels in breast cancer cells using MethSurv database. The highest levels of DNA methylation in patients with breast cancer were as follow: cg10062803 and cg14152613 of FABP4; cg06842886, cg14584085, and cg21978128 of ADIPOQ; cg07895576 and cg16827534 of PPARG; cg09427718, cg06772578, and cg08550435 of PPARGC1A; cg05345249 of CD36; cg16560077, cg01963870, cg27390443, cg27318635, cg03140190, and cg05898629 of CREBBP.
PPARGC1A

## Slide 6
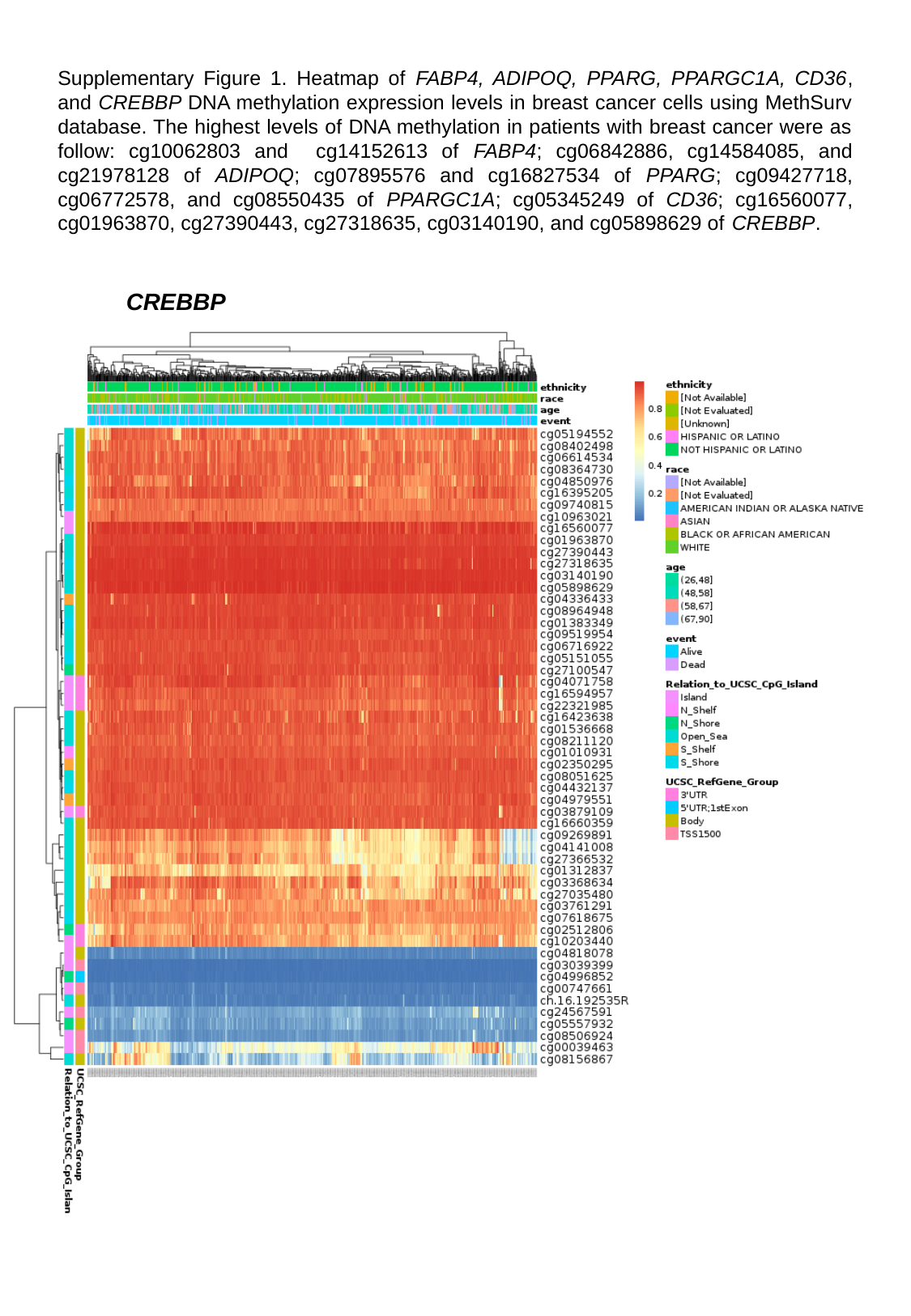

# Supplementary Figure 1. Heatmap of FABP4, ADIPOQ, PPARG, PPARGC1A, CD36, and CREBBP DNA methylation expression levels in breast cancer cells using MethSurv database. The highest levels of DNA methylation in patients with breast cancer were as follow: cg10062803 and cg14152613 of FABP4; cg06842886, cg14584085, and cg21978128 of ADIPOQ; cg07895576 and cg16827534 of PPARG; cg09427718, cg06772578, and cg08550435 of PPARGC1A; cg05345249 of CD36; cg16560077, cg01963870, cg27390443, cg27318635, cg03140190, and cg05898629 of CREBBP.
CREBBP

## Slide 7
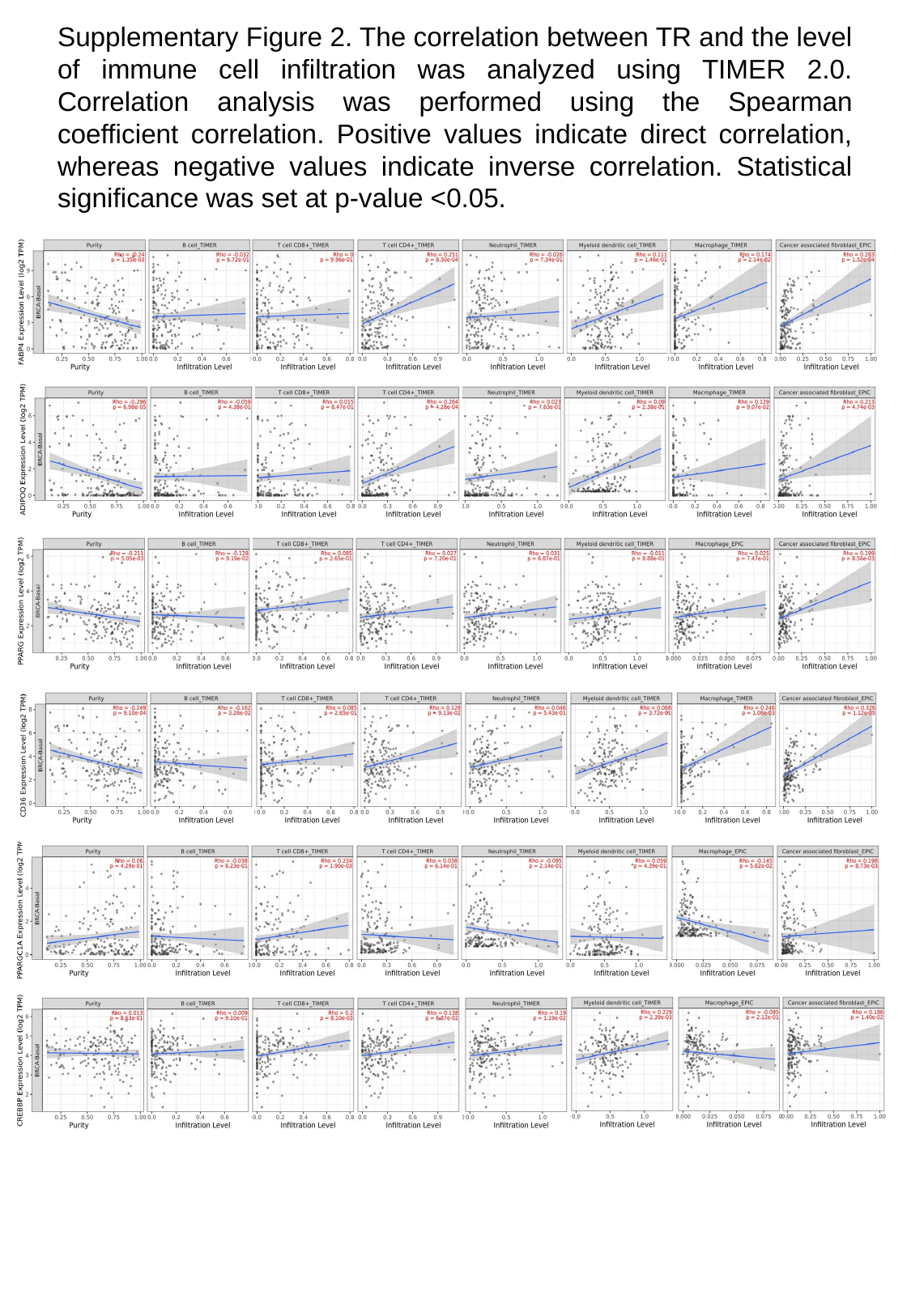

Supplementary Figure 2. The correlation between TR and the level of immune cell infiltration was analyzed using TIMER 2.0. Correlation analysis was performed using the Spearman coefficient correlation. Positive values indicate direct correlation, whereas negative values indicate inverse correlation. Statistical significance was set at p-value <0.05.
